# Redox Homeostasis in Muscular Dystrophies

**DOI:** 10.3390/cells10061364

**Published:** 2021-06-01

**Authors:** Nicola Mosca, Sara Petrillo, Sara Bortolani, Mauro Monforte, Enzo Ricci, Fiorella Piemonte, Giorgio Tasca

**Affiliations:** 1 Unità Operativa Complessa di Neurologia, Fondazione Policlinico Universitario A. Gemelli IRCCS, 00168 Roma, Italy; nicolamosca85@gmail.com (N.M.); s.bortolani@gmail.com (S.B.); mauro.monforte@gmail.com (M.M.); 2 Unit of Muscular and Neurodegenerative Diseases, Ospedale Pediatrico Bambino Gesù, IRCCS, 00146 Rome, Italy; sara.petrillo@opbg.net (S.P.); fiorella.piemonte@opbg.net (F.P.); 3 Istituto di Neurologia, Università Cattolica del Sacro Cuore, 00168 Roma, Italy

**Keywords:** muscular dystrophies, FSHD, oxidative stress, reactive oxygen species (ROS), antioxidants, Nrf2, inflammation

## Abstract

In recent years, growing evidence has suggested a prominent role of oxidative stress in the pathophysiology of several early- and adult-onset muscle disorders, although effective antioxidant treatments are still lacking. Oxidative stress causes cell damage by affecting protein function, membrane structure, lipid metabolism, and DNA integrity, thus interfering with skeletal muscle homeostasis and functionality. Some features related to oxidative stress, such as chronic inflammation, defective regeneration, and mitochondrial damage are shared among most muscular dystrophies, and Nrf2 has been shown to be a central player in antagonizing redox imbalance in several of these disorders. However, the exact mechanisms leading to overproduction of reactive oxygen species and deregulation in the cellular antioxidants system seem to be, to a large extent, disease-specific, and the clarification of these mechanisms in vivo in humans is the cornerstone for the development of targeted antioxidant therapies, which will require testing in appropriately designed clinical trials.

## 1. Introduction

The oxidative damage caused by an overproduction of reactive oxygen species (ROS) to biomolecules may affect skeletal muscle homeostasis and functionality, thus exacerbating pathological conditions in hereditary myopathies and muscular dystrophies [1]. Indeed, muscle degeneration, mitochondrial dysfunction, inflammation, and insufficient muscle regeneration are strictly linked to oxidative stress, which stems from the imbalance between ROS generation and protection provided by antioxidants. When such a disequilibrium of redox homeostasis occurs, the first intracellular defense line is represented by the activation of the transcription factor nuclear factor erythroid 2-related factor 2 (Nrf2), the master regulator of antioxidant response [2]. Many Nrf2 inducers have been reported to counteract oxidative stress in several physiological and pathological conditions, by upregulating the antioxidant defenses, inhibiting inflammation, and improving mitochondrial function [3,4].

Muscular dystrophies are a phenotypically and genotypically heterogeneous group of inherited muscular disorders with common histopathological features that include inflammation, degeneration, necrosis, and fibrosis. In addition to the pathogenic consequence arising from individual gene mutations, oxidative stress has been identified as one of the most relevant causes of muscle damage in these disorders [1,5,6].

This review summarizes the current knowledge about the role of oxidative stress in the pathogenesis of muscular dystrophies and highlights potential new targets for therapies.

## 2. What Is Oxidative Stress and How Can It Affect Muscles?

ROS are signaling molecules moderately and continuously produced by skeletal muscles as a consequence of their contractile activity and high mitochondrial O_2_ consumption [6]. The main source of ROS production is located in the cytosol through the activity of nicotinamide adenine dinucleotide phosphate (NADPH) oxidases (NOX enzymes), xanthine oxidase (XO), and nitric oxide synthase (NOS), and by the mitochondrial electron transport chain [1]. When ROS exceed the antioxidant buffering capacity of tissues, oxidative stress occurs [7]. The upregulation of the endogenous antioxidant system is the main response to ROS-induced stress, aiming at scavenging excessive ROS before they cause harm to tissues. ROS can be catalytically neutralized by antioxidant enzymes, including superoxide dismutase (SOD), catalase (CAT), glutathione peroxidase (GPx), and peroxiredoxins (PRDX), and by the action of the tripeptide glutathione (GSH) that directly buffers ROS [1]. High levels of these antioxidants are present in oxidative muscle fibers (type I and IIa in mice) [8,9], and their deficiency is a hallmark of oxidative stress. A secondary antioxidant defense line also exists in cells and is represented by the enzymes belonging to the inducible phase II (i.e., the glutathione *S*-transferase family enzymes, GST), which are activated by a number of different byproducts, such as xenobiotics, drugs, lipid peroxidation products, and chemical compounds (isothiocyanates and phenols) [10]. In addition, other enzymes, such as heme oxygenase-1 (HO-1), are responsible for the synthesis of nonenzymatic antioxidants present in the skeletal muscle (biliverdin and bilirubin) and play an important role in the regulation of heme catabolism [8]. Lastly, nicotinamide adenine dinucleotide phosphate quinone oxidoreductase 1 (NQO1) is critical in cellular adaptation to stress and is significantly elevated in skeletal muscles in response to aerobic exercise [9].

The balance between production of ROS and antioxidant defenses plays a fundamental role in maintaining redox homeostasis in muscle, while redox imbalance may exacerbate the progression of several muscle diseases. This is particularly true for chronic disorders, such as muscular dystrophies, characterized by the exposure to continuous damage (e.g., due to the lack of dystrophin) and an inappropriate response to injury (e.g., inflammation and profibrotic response).

**Figure 1:**
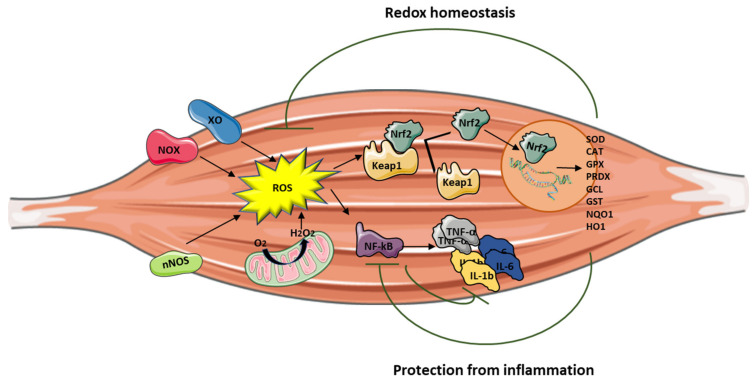
Central role of Nrf2 in protecting muscle from oxidative stress and inflammation. Abbreviations: XO: xanthine oxidase; NOS: nitric oxide synthase; NOX: nicotinamide adenine dinucleotide phosphate oxidases, ROS: reactive oxygen species; Nrf2: nuclear factor erythroid 2-related factor 2; Keap1: Kelch-like ECH-associated protein 1; NF-κB: nuclear factor kappa-light-chain-enhancer of activated B cells; TNF-α: tumor necrosis factor alpha, IL-6: interleukin-6, IL-1β: interleukin-1β; SOD: superoxide dismutase; CAT: catalase; GPx: glutathione peroxidase; PRDX: peroxiredoxin; GCL: glutamate cysteine ligase; GST: glutathione *S*-transferase; NQO1: nicotinamide adenine dinucleotide phosphate quinone oxidoreductase 1; HO1: heme oxygenase-1.

Although ROS have been reported to be mainly harmful, they also play a crucial role in cell signaling by activating Nrf2, which is in turn responsible for regulating the expression of more than 200 cytoprotective redox genes [11,12]. Nrf2 is a member of the basic leucine zipper (bZIP) family of transcription factors and is physiologically repressed through the binding to the protein Keap1 (Kelch-like ECH-associated protein 1) [12]. Upon exposure to ROS, Nrf2 is released from Keap1 and accumulates in the nucleus where it activates many antioxidant and anti-inflammatory target genes [12] (Figure 1). Nrf2 also modulates the NADPH oxidase enzyme activity, which has a central role in the pathophysiology of muscular dystrophies as source of ROS [13,14,15]. Thus, Nrf2 may have a critical role in the development of damage in muscular dystrophies, and its pharmacological activation can contribute to mitigate oxidative stress in muscles and to improve muscle performance [16,17].

## 3. The Role of Oxidative Stress in Muscle Inflammation

Inflammation can be triggered by an imbalance of cellular oxidative–antioxidant homeostasis. Usually, a moderate ROS production does not stimulate inflammation since ROS are mostly removed by cellular antioxidant systems. However, when ROS production, catalyzed by enzymes including NOX, COX-2, and XO, exceeds the threshold of redox balance, NF-κB is activated, thus triggering inflammation [18,19].

NF-κB is, therefore, the primary redox-sensitive signaling pathway of inflammation. The activation of protein kinase C (PKC) and NF-κB-induced kinase, a member of the MAPK family, is known to activate IKK, which phosphorylates the IκB subunit of the NF-κB complex and unleashes p50/p65 for nuclear binding [18,20]. As a result of NF-κB activation, proinflammatory cytokines, such as TNF-α, IL-1β, and IL-6, are produced by immune cells and/or damaged muscle tissues, thus further promoting the expression of adhesion molecules, including vascular cell adhesion molecule 1 (VCAM-1), cytokine-induced neutrophil chemoattractant-1 (CINC-1), and monocyte chemoattractant protein-1 (MCP-1), together with NOS upregulation [21]. Moreover, the NO production contributes to increase blood flow and the chemotactic effects of adhesion molecules, facilitating the infiltration of inflammatory cells into the affected area.

Mitochondria are also involved in the inflammatory process, and high levels of proinflammatory cytokines affect mitochondrial ROS generation and metabolic function. PGC-1α, which is a key regulator of muscle metabolism and mitochondrial biogenesis, also plays a role as an anti-inflammatory agent. Studies performed on PGC-1α KO mice models demonstrated high basal mRNA expression of TNF-α and IL-6 in skeletal muscle and in serum, showing that PGC-1α is able to effectively modulate local and systemic inflammation [22]. In addition, mice overexpressing PGC-1α showed low expression of TNF-α and IL-6 [23], suggesting that PGC-1α may play a protective role in the inflammatory response by reducing the generation of proinflammatory cytokines.

Nrf2 activation can attenuate inflammation by modulating the expression of NF-κB [16,24] (Figure 1). Thus, Nrf2 and NF-κB are interrelated in the inflammatory response [25]. Nrf2 exerts its anti-inflammatory function via the activation of HO-1 that catalyzes the degradation of heme into carbon monoxide (CO), free iron (Fe^2+^), and biliverdin, before being converted to the antioxidant bilirubin [25,26,27]. Muscle damage and inflammation worsened after ablation of HO-1 in the mdx mouse [28], while pharmacological induction of HO-1 exerted a protective effect on skeletal muscle through the inhibition of NF-κB [29].

It is important to note that Nrf2 binds DNA in proximity of the genes encoding IL-1β and IL-18 to modulate their transcription [30], thereby supporting its direct inhibitory effect on NOD-like receptor protein 3 (NLRP3) inflammasome priming.

## 4. Oxidative Stress in Early Onset Myopathies

Early-onset myopathies are inherited muscle diseases occurring during infancy or early childhood. These can be further classified into congenital muscular dystrophies (CMDs) and congenital myopathies (CMs) [31,32]. They are typically associated with muscle weakness and hypotonia, delayed motor development, and difficult or absent ambulation, sometimes with orthopedic complications, respiratory and cardiac failure, and, in the most severe forms, premature death [31].

CMDs and CMs have been classified on the basis of their major muscle morphological features. Patients with CMDs present dystrophic lesions on muscle biopsies, with or without necrotic fibers and regeneration, whereas, in congenital myopathies, muscles are generally not dystrophic but show characteristic structural changes in the internal fiber architecture [33]. CMDs and CMs share several pathophysiological pathways, involving proteins essential for the embryonic muscle development, the complex architectural structure of muscle fibers, the excitation/contraction coupling, and the redox regulation system [33].

The deregulation of redox homeostasis is a mechanism implicated in the pathogenesis of early-onset myopathies, and significant progress has been reached in the understanding of these diseases through the analysis of oxidative-mediated processes [34,35,36,37]. Proteins involved in redox regulation represent hallmarks and potential therapeutic targets in several early-onset myopathies, including RYR1- and SEPN1-related myopathies, and in Duchenne muscular dystrophy (DMD) [38,39,40,41].

RYR1-related myopathies are a group of skeletal muscle disorders caused by mutations in the ryanodine receptor gene, *RYR1* [42]. They are clinically characterized by a wide spectrum of symptoms ranging from slowly progressive hip and axial muscle proximal weakness, to malignant hyperthermia [43,44]. RYR1, also known as skeletal muscle calcium release channel, is an essential component of the excitation–contraction coupling apparatus and contains many redox-sensitive reactive cysteines, strongly involved in calcium handling [45,46,47,48,49]. Mitochondrial dysfunction and redox imbalance have been associated with *RYR1* primary defects, thus emerging as promising therapeutic targets for the disease. The use of N-acetylcysteine (NAC), the precursor of the antioxidant glutathione (GSH), improved muscle function and restored myotube phenotype in *RYR1* mutated cell cultures and in the Y522S mouse model, opening the way to clinical trials in patients [50,51,52,53].

Defects in redox homeostasis have also been described in SEPN1-related myopathy, an inherited muscle disease caused by mutations in gene encoding selenoprotein-N (*SELENON*), which displays clinical and pathological overlap with RYR1-related myopathies [39]. Selenoproteins are a family of proteins containing several selenocysteine residues involved in modulating redox calcium homeostasis and protecting against oxidative stress [39]. In addition, the *SEPN1* gene promoter contains sequences for NF-κB, the primary redox-sensitive signaling pathway in inflammation [39]. *SEPN1*-mutated patients often show a severe weakness of neck and trunk muscle, leading to scoliosis, spinal rigidity, and life-threatening respiratory insufficiency [54]. Limited motility and body rigidity after forced swimming test were reported in Sepn1-deficient mouse models [55], and increased protein oxidation, abrogated after pretreating cells with NAC, was described in myoblasts primary cultures from patients [56], leading to NAC clinical trials (SELNAC, clinicaltrials.gov, identifier: NCT02505087).

Increasing evidence shows a central role for oxidative stress in DMD and Becker muscular dystrophy (BMD), the most common muscular dystrophies in childhood. DMD is a severe X-linked recessive neuromuscular disorder affecting one in 3600 boys [57]. Muscle degeneration results in weakness, delayed motor milestones, and, eventually, loss of ambulation. DMD is caused by mutations in the gene encoding for dystrophin, a membrane protein whose absence is responsible for increased susceptibility to damage, with myofiber necrosis and secondary inflammation as a result of skeletal muscle contraction [57,58,59]. Dystrophin is also expressed in cardiomyocytes, and patients also develop cardiomyopathy. Mutations in the same gene may also result in a mildly defective dystrophin protein production, clinically corresponding to BMD, characterized by a less severe phenotype [57].

Oxidative stress makes the dystrophin-deficient heart and skeletal muscle highly susceptible to injury, exacerbating the pathological features of the disease [60,61,62,63,64]. NOX is the main source of ROS in dystrophin-deficient models, contributing to enhanced Ca^2+^ influx and activation of the Src kinase [65]. Furthermore, decreased levels of the antioxidant GSH, with a concomitant increase of its oxidized form GSSG and a reduced activity of the protective enzyme GPX, have been found in dystrophic muscles and in peripheral blood of patients with DMD [66,67]. Notably, preclinical studies using antioxidant drugs have evidenced beneficial effects on dystrophin-deficient mouse models, such as reduced muscle damage, decreased necrosis, inflammation, and fibrosis [68,69,70,71,72,73].

## 5. Oxidative Stress in Adult-Onset Muscular Dystrophies

Adult-onset muscular dystrophies are inherited disorders with different severity, age of onset, and type of muscle involvement. Among them, the most prevalent are myotonic dystrophy type 1 (Steinert disease), facioscapulohumeral muscular dystrophy, and limb girdle muscular dystrophies.

### 5.1. Facioscapulohumeral Muscular Dystrophy

Facioscapulohumeral muscular dystrophy (FSHD) is one of the most common forms of muscular dystrophy. It is characterized by a distinctive pattern of skeletal muscle weakness and a wide spectrum of disease severity. FSHD type 1 was mapped to chromosome 4q35 and is associated with a contraction in a macrosatellite repeat array known as D4Z4 [74,75]. This contraction, together with a polyadenylation signal distal to the repeats, allows the stable transcription of the *DUX4* retrogene that is normally repressed in somatic tissues including skeletal muscle. The DUX4 protein is highly toxic and can induce apoptosis, through activation of caspase-3. Moreover, DUX4 expression in skeletal muscle can also lead to activation of different genes involved in atrophy, protein degradation, and innate immunity, and it negatively regulates myogenesis [76,77].

**Figure 2:**
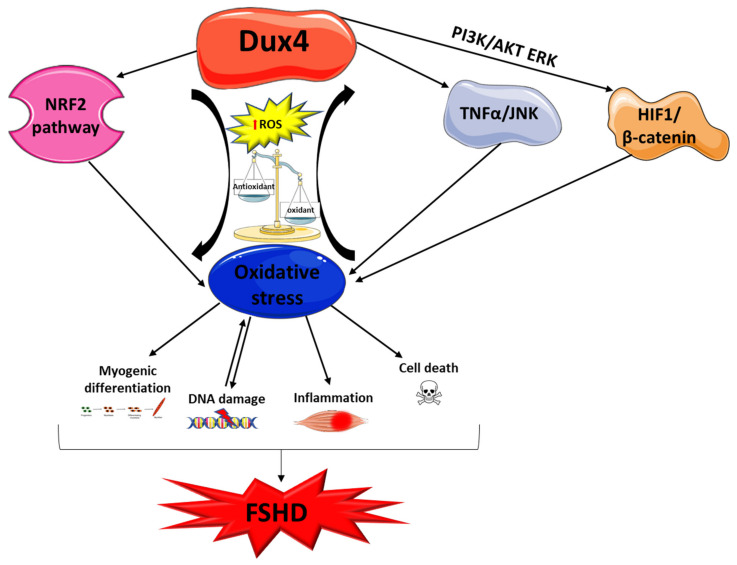
Interplay between DUX4 and oxidative stress in FSHD. This schematic highlights the role of DUX4 in oxidative stress induction during FSHD development and progression. Abbreviations: DUX4: double homeobox 4; Nrf2: nuclear factor erythroid 2-related factor 2; ROS: reactive oxygen species; PI3K: phosphatidylinositol 3-kinase; ATK: protein kinase B; ERK: mitogen-activated protein kinase, TNF-α: tumor necrosis factor alpha; JNK: c-Jun N-terminal kinase; HIF-1: hypoxia-inducible factor 1.

Since DUX4 is a central player in FSHD pathogenesis, different authors focused their attention on its role in the development of oxidative stress (Figure 2). Bosnakovski and colleagues showed that DUX4 alters the expression of genes implicated in redox balance and enhances the sensitivity of C2C12 myoblasts to pro-oxidant compounds, while antioxidant treatment reduced DUX4 toxicity [78,79]. Interestingly, oxidative stress induced by DUX4 is a direct cause of DNA damage, and both DNA damage and oxidative stress can also affect the myogenic differentiation process contributing to aberrant myotube formation [80]. Noteworthy is that DNA damage caused by moderate doses of oxidants is efficiently repaired in FSHD myoblasts, suggesting the capacity of handling oxidative stress up to a certain level [81]. On the other hand, the treatment of patient-derived induced pluripotent stem cells (iPSC) with H_2_O_2_ significantly increases the transcription of *DUX4* and its targets. In this case, the DNA damage induced by oxidative stress occurs prior to DUX4 increase and this effect seems to be mediated by ataxia telangiectasia mutated serine/threonine kinase (ATM) activation [82]. This evidence shows that a feedback loop regulation may occur between DUX4 and oxidative stress contributing to muscle damage in FSHD. Furthermore, DUX4 can activate TNF-α and JNK pathways, increasing susceptibility to oxidative stress-induced cell death, as well as regulate hypoxia-inducible factor 1α (*HIF1A*) expression, which, upon interacting with β-catenin, inhibits cell proliferation and induces transcription of hypoxic response genes [83]. Interestingly, it is known that high levels of ROS can induce HIF-1α stabilization via PI3K/ATK and ERK pathways [84].

DUX4 is also able to modulate the Nrf2-mediated oxidative stress response pathway. Sharma and colleagues, in a transcriptomic study conducted on human rhabdomyosarcoma cells overexpressing DUX4, identified 31 out of the 86 transcripts known to function in the Nrf2 pathway as differentially expressed, with the majority of these changes potentially inducing or contributing to oxidative stress [85]. The study confirms the link between Nrf2 and DUX4, and it further highlights the role of DUX4 in the induction of oxidative stress.

Lastly, DUX4 seems to be negatively involved in the plasma membrane repair pathways. Indeed, Bittel and colleagues demonstrated, using a cellular and animal model, that DUX4 inhibition, as well as antioxidant treatment, is able to improve plasma membrane repair by reducing mitochondrial ROS levels [86].

Further evidence coming from earlier studies supports additional roles for oxidative stress in the development of muscle wasting in FSHD. Winokur and colleagues demonstrated that FSHD myoblasts are sensitive to oxidative stress upon exposure to paraquat and that this susceptibility is no longer seen upon differentiation to myotubes [87]. They also showed an alteration of several genes involved in oxidative stress, particularly a glutathione *S*-transferase theta-2 (*GSTT2*) downregulation, suggesting a reduced capacity to buffer oxidative stress [87]. Furthermore, ANT1, one of the most abundant mitochondrial inner membrane proteins, is reported to be increased in FSHD muscles with the highest level of protein detected in the clinically unaffected muscles, suggesting that this upregulation may be an early phenomenon in FSHD pathogenesis. Overexpression of ANT1 is associated with altered expression of different enzymes related to oxidative stress, such as SOD1, GST, and thioredoxin peroxidase [88,89,90]. Moreover, increased expression of ANT1 leads to recruitment of the IκBa/NF-κB complex into mitochondria, with a decrease in nuclear NF-κB DNA-binding activity. In this scenario, genes with antioxidant and antiapoptotic activity are downregulated and, consequently, muscle cells are sensitized to oxidative stress and apoptosis [91]. Another potential link between oxidative stress and FSHD muscle wasting is the mitochondria ultrastructure alteration in affected muscles. In FSHD, the mitochondrial ultrastructure of myofibers is altered, and mitochondria accumulate. In these regions, mitochondria present badly formed cristae or inner and outer membrane separation. These structural alterations are associated with mitochondrial dysfunctions, such as reduced COX activity and ATP production, as well as oxidative stress imbalance [92]. More recently, Banerji and colleagues, using a dynamic transcriptomic analysis that combines high-throughput time course imaging and transcriptomics, showed a downregulation of the ERRα/PGC1α pathway in immortalized FSHD human myoblast cell lines. PGC1α, which is critical for mitochondria biogenesis, is directly involved in defense against oxidative stress, upregulating different antioxidant enzymes such as Manganese superoxide dismutase (MnSOD) [93].

Lastly, it is intriguing that inflammation seems to be involved in the development of muscle damage in FSHD with a progression that can be followed using muscle imaging [94,95]. Although its role is not completely understood, in vivo evidence suggests that it constitutes an active process in muscles undergoing early damage [96] and significantly involves cytokines and mediators of the innate immunity arm [97]. The inappropriate expression of cancer testis and other autoantigens has been evoked as a possible trigger of the inflammatory response, but conclusive evidence is currently lacking. The possible interplay between inflammation and oxidative stress in FSHD also needs clarification, as preliminary evidence suggests a redox imbalance in muscles showing signs of early damage. In these muscles, proteomic analysis of the interstitial fluid analyzed by mass spectrometry identified a downregulation of SOD1 and upregulation of GPx3 and CAT [98]. Taken together, these data suggest that targeting specific players in these pathways may serve as a therapeutic approach to reduce oxidative stress in FSHD.

### 5.2. Myotonic Dystrophy Type 1

Oxidative stress is also a player in the pathophysiology of myotonic dystrophy type 1 (DM1). DM1 is the most common form of muscular dystrophy in the adult and is caused by an expansion of CTG repeats in the 3′-untranslated region of the myotonic dystrophy protein kinase (*DMPK*) gene [99]. In 1995, Ihara and colleagues proposed a role for oxidative stress in DM1 on the basis of the discovery in patients’ blood of an increased level of free radicals and lipid peroxides, as well as a decrease in antioxidants such as α-tocopherol, coenzyme Q10, selenium, and albumin [100]. Usaki and colleagues, using the C2C12 cell model, demonstrated that the susceptibility to oxidative stress is CTG-repeat number-dependent, suggesting that it could be involved in the pathogenesis of the disease. Furthermore, using the same model, they found that the induction of apoptosis by oxidative stress is related to the activation of the SAPK/JNK pathway and the inhibition of the ERK pathway [101]. DM1 is a multisystemic disorder with an involvement also of the central nervous system. In a mouse model of DM1, the chronic administration of methylphenidate (MPH) was able to reduce the reactive microglia and proinflammatory cytokine IL-1β levels [102]. The treatment increased the *NRF2* gene expression in the hippocampus of the DM1 mice by modulating the brain-derived neurotrophic factor (BDNF) levels, which regulates Nrf2 nuclear translocation [103]. These findings support a role for Nrf2 in mediating the reduction of oxidative stress and inflammation in DM1 and address the pharmacological Nrf2 induction as a promising therapy in this disease [104].

Moreover, evidence indicates that mitochondrial dysfunction is involved in the pathophysiology of DM1. In this regard, signs of mitochondrial alteration in muscle biopsy and oxidative stress markers have been detected in different DM1 patient cohorts [105,106]. Lastly, recent studies focused the attention on antioxidant system deregulation showing the downregulation of several proteins such as GPx, Gst, and GSH in DM1 patients [107,108].

### 5.3. Limb Girdle Muscular Dystrophies

Limb girdle muscular dystrophies (LGMDs) constitute a group of genetic disorders characterized by progressive weakness and wasting of the proximal limb muscles, with onset by definition after the acquisition of autonomous ambulation. Several studies showed the involvement of oxidative stress in calpainopathy (LGMDR1, previously named LGMD2A). LGMDR1 is caused by mutations in the gene encoding calpain-3 (*CAPN3*), a non-lysosomal calcium-dependent cysteine protease, and *Capn3* KO mice show abnormalities in mitochondrial structure, distribution, and function, suggesting that energy production deficits, along with increased oxidative stress, are pathogenic features of LGMDR1 [109]. Moreover, oxidative and nitrosative stress occurring in LGMDR1 activate different pathways such as the NF-κB one that is involved in muscle wasting [110].

Redox imbalance has also been detected in LGMDR1 patients [111], where reductions in antioxidant defense mechanisms (SOD1 and Nrf2), coupled with increased lipid peroxidation and protein ubiquitination, were found. The redox imbalance primarily affected nonmitochondrial compartments, since the enzyme activities of citrate synthase, cytochrome c oxidase, and complex I + III were comparable to controls.

Lastly, a relevant upregulation of oxidative stress and NF-κB signaling has also been identified in dysferlinopathy or LGMDR2. Analyses on human precursor and differentiated cultured muscle cells demonstrated that a reduction in dysferlin induces oxidative stress with the mitochondria as a source of ROS [112].

## 6. Potential Targets for Therapy

**Figure 3:**
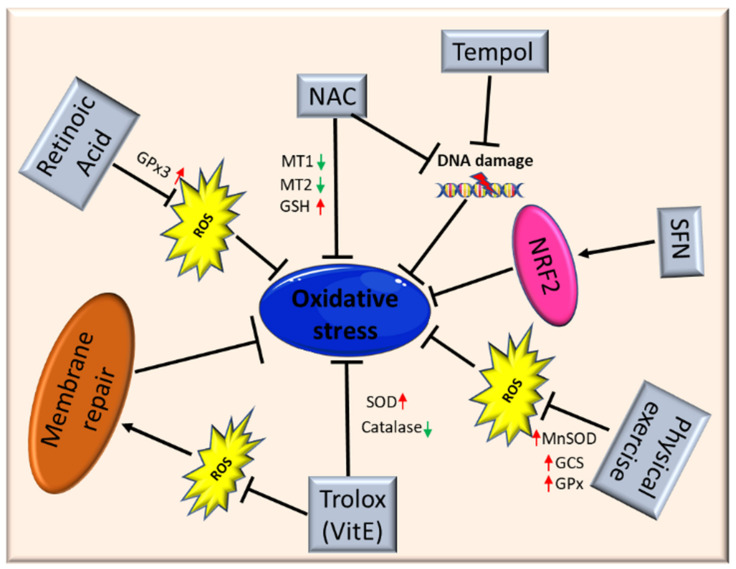
Schematic illustration of antioxidant therapy targets in muscular dystrophies. Abbreviations: ROS: reactive oxygen species; NAC: *N*-acetylcysteine; MT1: metallothionein 1; MT2: metallothionein 2; GSH: glutathione; SFN: isothiocyanate sulforaphane; Nrf2: nuclear factor erythroid 2-related factor 2; MnSOD: manganese superoxide dismutase; GCS: γ-glutamylcysteine synthetase; GPx: glutathione peroxidase; SOD: superoxide dismutase.

As oxidative stress is involved in muscle homeostasis and functionality, antioxidant treatment may be a potential therapeutic option, alone or as an adjuvant, to treat myopathies and muscular dystrophies. To date, different studies have shown the ability of antioxidants to improve muscle health, reducing ROS levels through the modulation of different genes involved in oxidative stress response (Figure 3).

El Haddad and colleagues reported that treatment with retinoic acid, a metabolite of vitamin A, reduced ROS level in human myoblasts derived from both healthy subjects and FSHD patients and improved cell survival in transplantation assays. Moreover, they underlined the molecular mechanism involved, further demonstrating that retinoic acid is able to induce expression and increase activity of GPx3 [113].

Another study demonstrated that treatment with tempol, a powerful antioxidant, of DUX4-transfected myoblasts, as well as myoblasts derived from FSHD patients, efficiently reduced the level of ROS and DNA breaks.

Similar findings were obtained using NAC [80]. Moreover, deferoxamine (DFX) was able to improve the antioxidant effects of NAC on primary cultures from mdx mice mainly reducing H_2_O_2_ production and NF-κB levels [71]. NAC treatment also decreased oxidative stress-responsive genes metallothionein1 and 2 (*Mt1* and *Mt2*), ameliorating myopathic phenotypes in a mouse model of GNE myopathy, one of the most prevalent distal myopathies [114].

Vitamin E is commonly used as an antioxidant to limit oxidative damage and inflammatory disease. Bittel and colleagues reported that treatment with Trolox, a water-soluble analogue of vitamin E, significantly improved membrane repair capacity by reducing mitochondrial ROS levels in FSHD myoblasts [86]. Furthermore, vitamin E is able to significantly increase the level of SOD and decrease the catalase level in mdx mice, suggesting that it may contribute to counteract the deleterious effects of oxidative damage, including lipid peroxidation and free-radical generation [68]. Bosnakovski and colleagues identified 52 compounds that can inhibit DUX4-induced toxicity in FSHD myoblasts. Two-thirds of these compounds have been shown to reduce oxidative stress in FSHD muscle, along with slowing disease processes [79].

Several studies also highlighted the role of physical exercise to counteract oxidative stress in muscle. Increasing evidence suggests that the continued presence of a small stimulus, such as low concentrations of ROS, can induce a compensatory increase of antioxidant defenses. For instance, Gomez-Cabrera and colleagues demonstrated that exhaustive exercise in animals resulted in reduced oxidative damage due to the upregulation of endogenous antioxidant enzymes such as MnSOD, GPx, and γ-glutamylcysteine synthetase (GCS), along with an enhanced NF-κB pathway [115]. Therefore, physical exercise should be considered beneficial in patients with muscular dystrophies.

It is important to underline once again that Nrf2 is the primary regulator of the endogenous antioxidant response, and that Nrf2 induction is a more efficient alternative to the use of a single antioxidant. Indeed, unlike other strategies, targeting Nrf2 activation has the potential to simultaneously modulate separate pathological features and amplify therapeutic benefits in muscular dystrophies [2]. Nrf2 may ensure a durable benefit in chronic disease states via upstream regulation of several antioxidant and anti-inflammatory pathways. Many Nrf2 activators have been identified, some of those are either already in clinical practice or tested in interventional trials (e.g., the fumaric acid esters, oltipraz and ursodiol) [116,117,118]. For instance, the use of the isothiocyanate sulforaphane (SFN), a well-known Nrf2 inducer [119], was able to attenuate muscle inflammation and fibrosis in mdx mice [16]. A recent study further confirmed the importance of targeting Nrf2 to amplify the therapeutic benefits and open the way to new drugs for treating chronic muscle diseases [2].

However, despite the amount of preclinical evidence about the central role of oxidative stress in development of muscle damage in muscular dystrophies, early clinical trials using antioxidants such as nicotinamide (vitamin B) and tocopherols (vitamin E) have so far failed to bring significant clinical benefits [37]. Nevertheless, a double-blind randomized controlled clinical trial with antioxidant supplementation (vitamin C, vitamin E, zinc gluconate, and selenium) in FSHD patients was shown to minimize some adverse effects related to oxidative damage (particularly lipid peroxidation) resulting in an improvement of muscle strength [120]. Furthermore, more recently, Sitzia and colleagues, in a single-center randomized double-blind placebo-controlled study, reported clinical benefit on 29 patients with DMD, FSHD, or LGMD after the supplementation with flavonoids/omega3 (FLAVOMEGA) [121].

## 7. Conclusions

Evidence coming from the literature suggests that oxidative stress is an important modulator of skeletal muscle homeostasis and functionality. Because of their contractile activity, as well as their high oxygen consumption, and metabolic rate, skeletal muscles continuously produce moderate levels of ROS. Under normal conditions, there is a balance between pro-oxidative and antioxidative factors to allow the proper functioning of metabolic pathways, as well as cellular integrity [6,122,123]. If this balance is disrupted, the accumulation of oxidative agents may lead to cellular dysfunction and death, resulting in chronic tissue degeneration and inflammation [124]. As discussed, oxidative stress is one of the most relevant causes of muscle damage, and it is not surprising that an impairment of redox homeostasis has been evidenced in several muscle disorders.

Since redox homeostasis is drug-targetable, the implementation of pharmacological trials with antioxidants can pave the way for novel promising preclinical studies. Nevertheless, many questions need further investigation, such as the exact mechanism underlying oxidative stress in patients with muscle disorders and the effective benefits of antioxidant supplementation in specific forms of muscular dystrophies.

## Data Availability

Not applicable.
